# Mendel,MD: a user-friendly online program for clinical exome analysis

**DOI:** 10.1186/1471-2105-16-S8-A2

**Published:** 2015-04-30

**Authors:** Raony Guimarães Corrêa Do Carmo Lisboa Cardenas, Natália Duarte Linhares, Sérgio D Junho Pena

**Affiliations:** 1 Laboratory of Clinical Genomics, Universidade Federal de Minas Gerais, Belo Horizonte, Brazil

## Background

With the advent of next-generation methodology, sequencing of the whole exome of a patient has become economically viable for clinical diagnosis of genetic diseases, including complex and rare ones. The strategy for identification of the pathogenic variant is complex, since in every exome there are 40 to 50 thousand nucleotide variants in comparison with the reference human genome. To simplify this procedure, computational filters that sequentially eliminate common and synonym variations, reducing the size of the total sample, should be used. After identifying pathogenic variants, laboratory confirmation should be carried out, for instance by traditional Sanger sequencing, to reach a definitive diagnosis.

The bioinformatics challenge is that the software has to be efficient and sophisticated from the computational point-of-view and, at the same time, simple and friendly to be used by clinicians. To address this matter, Mendel,MD was developed as a free and open-source tool that can be downloaded, installed and executed locally by any laboratory in the world with aim to analyze exomic data from their patients.

## Results

After submission of a standardized file with the exome information (VCF file) into the system, annotation with different methods and tools is done, preceded by calculation of metrics with the information generated. The information about the mean of coverage and quality for all the variants of each individual is presented. Those values are used when defining thresholds for the parameters in the next implemented method which is called Filter Analysis.

Filter Analysis is a method which combines different annotations, databases and scores of pathogenicity allowing to reduce the number of variants and genes of each clinical case from thousands of candidates to only a few dozens. We claim that the final list of genes should always be investigated by doctors and researchers in the search for good candidates causing mutations taking into consideration each specific clinical case.

In order to integrate into the results the possibility of considering different models of inheritance (recessive, compound heterozygous, dominant and X-linked) the Family Analysis method was developed. It enables the search for compound heterozygous variants (the mutation which comes from both parents) and de novo variants in exomes from trios, quartets or even a larger number of individuals from a certain family.

The ultimate method developed in our tool is Pathway Analysis and it can be used to investigate variants and genes grouped by each pathway in KEGG. To test the method we used data from two different disorders Hurler Syndrome and Hunter Syndrome respectively, which, although caused by mutations in two different genes (IDUA and IDS) are both members of the same pathway category (glycosaminoglycan degradation).

The tool was validated with data from 15 different clinical cases submitted from specialized laboratories from different countries. It was consistently possible to identify a very short list of causal gene candidates, which included the correct diagnosis in all cases.

## Conclusions

Mendel,MD is an efficient, secure and reliable software in exploration of variants from exome data of patients with Mendelian disorders, sophisticated from the bioinformatics perspective and yet simple enough to be used by doctors and scientists to quickly analyze genomic data.

